# A co-expression network for differentially expressed genes in bladder cancer and a risk score model for predicting survival

**DOI:** 10.1186/s41065-019-0100-1

**Published:** 2019-07-09

**Authors:** Zihao Chen, Guojun Liu, Aslam Hossain, Irina G. Danilova, Mikhail A. Bolkov, Guoqing Liu, Irina A. Tuzankina, Wanlong Tan

**Affiliations:** 1 Department of Urology, Nanfang Hospital, Southern Medical University, Guangzhou, 510515 China; 2 Institute of Natural Sciences and Mathematics, Ural Federal University, Ekaterinburg, 620000 Russia; 3 Institute of Chemical Engineering, Ural Federal University, Ekaterinburg, 620000 Russia; 4 Institute of Immunology and Physiology, Ural Branch of the Russian Academy of Sciences, Ekaterinburg, 620000 Russia; 5 School of Life Science and Technology, Inner Mongolia University of Science and Technology, Baotou, 014010 China

**Keywords:** Bladder cancer, WGCNA, TPST1, P3H4, Risk score model

## Abstract

**Background:**

Urothelial bladder cancer (BLCA) is one of the most common internal malignancies worldwide with poor prognosis. This study aims to explore effective prognostic biomarkers and construct a prognostic risk score model for patients with BLCA.

**Methods:**

Weighted gene co-expression network analysis (WGCNA) was used for identifying the co-expression module related to the pathological stage of BLCA based on the RNA-Seq data retrieved from The Cancer Genome Atlas database. Prognostic biomarkers screened by Cox proportional hazard regression model and random forest were used to construct a risk score model that can predict the prognosis of patients with BLCA. The GSE13507 dataset was used as the independent testing dataset to test the performance of the risk score model in predicting the prognosis of patients with BLCA.

**Results:**

WGCNA identified seven co-expression modules, in which the brown module consisted of 77 genes was most significantly correlated with the pathological stage of BLCA. Cox proportional hazard regression model and random forest identified TPST1 and P3H4 as prognostic biomarkers. Elevated TPST1 and P3H4 expressions were associated with the high pathological stage and worse survival. The risk score model based on the expression level of TPST1 and P3H4 outperformed pathological stage indicators and previously proposed prognostic models.

**Conclusion:**

The gene co-expression network-based study could provide additional insight into the tumorigenesis and progression of BLCA, and our proposed risk score model may aid physicians in the assessment of the prognosis of patients with BLCA.

**Electronic supplementary material:**

The online version of this article (10.1186/s41065-019-0100-1) contains supplementary material, which is available to authorized users.

## Background

Urothelial bladder cancer (BLCA) is one of the most prevalent cancers worldwide. According to the report of the Chinese National Cancer Center, 80,500 Chinese were diagnosed with BLCA and 32,900 cases died during 2015 [1]. Currently, it is a challenging issue to predict the prognosis of BLCA patients since the options of the treatment are limited [2, 3]. The prognostic factors that can be used by physicians to predict the cancer-specific or overall survival are pathological grade and stage, Tumor Node Metastasis (TNM) staging system, number or size of tumors, and presence of carcinoma in situ [4–6], where pathological stage and TNM staging system represent the simplest, fastest and most commonly used prediction tools. Recently, it has been reported that traditional prediction factors are less accurate at prediction than prediction models incorporated molecular markers [7, 8].

The high-throughput sequencing methods along with its improved sequencing accuracy and decreased costs have greatly influenced the application of medical biomarker or signature in the cancer prognosis, prediction of recurrence, monitoring drug response, and developing targeted therapies [7, 9–11]. Although considerable progress has been made in recent years in identifying the molecular markers of disease and the development of multifactorial tools that predict the prognosis of BLCA [7–9], there are few qualified biomarkers are currently available to apply in BLCA prognostic models. It is thus imperative to identify and validate molecular biomarkers and incorporate them into multivariable predictive tools.

The Cancer Genome Atlas (TCGA) is a large integrated collection of clinical information and gene sequencing data, which allows to analysis in a systematic way on underlying molecular mechanisms of various cancers. A growing number of tumor sample datasets in the TCGA project enhance the statistical power and the ability to detect molecular defects in cancers. In addition, the latest progress on integrated multi-omics analyses had shed the new insight on cancer genomic [12–17]. Weighted gene co-expression network analysis (WGCNA), a systems biology algorithm, is extensively used in cancer, genetics of species, and other complex diseases research [18]. WGCNA can cluster functionally correlated genes into separate modules that provide the information on hub nodes based on the variability in the RNA-Seq and microarray data among biological samples. The modularity of the gene co-expression network allows us to investigate its components independently to further investigate network structures, biological processes, candidate biomarkers. Moreover, modules are more stable than individual genes, because the overall function of a module can remain the same while individual gene expression can be replaced or changed by other genes with similar redundant functions. Functional modules can, therefore, reveal more effectively the consistent differences during BLCA tumorigenesis and progression.

In this study, WGCNA was performed on the BLCA gene expression data retrieved from the TCGA data portal to identify gene co-expression modules associated with pathological stage and investigate the underlying hub genes. Furthermore, we identified prognostic-related biomarkers by performing Cox regression analysis and random forest and incorporated them into a risk score model for estimation of BLCA prognosis.

## Methods

### Data acquisition and pre-processing

The RNA-Seq raw count expression profile, Fragments Per Kilobase Million (FPKM) normalized expression profile, and clinical data of 414 BLCA samples and corresponding 19 healthy controls were respectively achieved from the TCGA data portal (https://cancergenome.nih.gov/). Raw count expression data of 414 BLCA samples and corresponding 19 healthy controls were used for differential expression analysis. After eliminating six recurrent samples and four samples without pathological stage information, the FPKM normalized gene expression data of 404 patients with BLCA was used for WGCNA analysis. The microarray gene expression profile and related clinical data of GSE13507 were obtained from Gene Expression Omnibus (GEO) and used to further validate our results (https://www.ncbi.nlm.nih.gov). The baseline characteristics of TCGA dataset and GSE13507 dataset were shown in Additional file 1: Table S1 and Additional file 2: Table S2.

### Screening for differentially expressed genes (DEGs) and enrichment analysis

To ensure the reproducibility and consistency of DEGs, we used “limma”, “edgeR”, and “DESeq” R packages to screen DEGs between tumor and normal samples, with |log2 Fold Change| > 1 and adjusted *P*-value < 0.05 as the cut-off values [19–22]^.^ For “limma” package, we used “voom” function with “quantile” parameter to normalize the expression level and then used “lmFit” followed by “eBayes” functions for fitting. For “edgeR”, we used “calcNormFactors” function to normalize the expression values and then used “exactTest” function to fit a negative binomial distribution of Trimmed Mean of M values (TMM) normalized counts. For “DESeq”, we used “estimateSizeFactors” followed by “counts” function to normalize the expression values. We then used the “nbinomTest” function to fit a negative binomial distribution of the scale factor normalized expression level.

Moreover, the Gene Ontology (GO) and Kyoto Encyclopedia of Genes and Genomes (KEGG) enrichment analyses for DEGs were performed, with Benjamini and Hochberg (BH) adjusted *P*-value < 0.05 as the cut-off value [23].

### WGCNA and protein-protein interactions

WGCNA was performed on DEGs to construct scale-free gene co-expression networks, with *min-ModuleSize* of 20 and *mergeCutHeight* of 0.25 [24]. An appropriate soft-threshold power was selected according to standard scale-free distribution. ﻿The Intramodular Connectivity was used to define the most highly connected hub gene in a module [24]. The co-expression network of genes within the pathological stage-related module was visualized with Cytoscape software (version 3.5.1.). The protein-protein interactions (PPIs) data of genes within the pathological stage-related module were retrieved from the STRING database (http://string-db.org/).

### Construction of prognostic rick score model

﻿We assessed the independent prognostic value of genes in the co-expression module associated with pathological stage using the univariate Cox proportional hazard regression model [25]. Subsequently, the analyses of random forest (RF) and multivariate Cox proportional hazard models were performed on the genes obtained by the previous step to select optimal prognostic biomarker combinations [26]. The biomarkers were further used to develop the risk score model and the formula of risk score is defined as follows:

$$ Risk\kern0.5em score=\kern0.5em \sum \limits_i^n{\beta}_i\kern0.5em \ast \kern0.5em x{}_i $$

where *β*_*i*_ indicates the coefficient for each gene and *x*_*i*_ indicates the z-score transformed relative expression value of each gene. Patients samples were divided into high and low-risk groups based on the median cutoff of the risk score and their survival difference was compared with Kaplan-Meier (K-M) survival analysis. The area under the ROC curve (AUC) was used to estimate the performance of the risk score model in the TCGA dataset and GSE13507 dataset. In addition, we assess the performance of the risk score model in an independent microarray dataset (GSE13507).

A nomogram was constructed to estimate one, three and five-year survival rate of patients with BLCA.

### Statistical analysis

All statistical analysis was performed using R statistical software (https://www.r-project.org/, v3.4.2). The correlation between biomarker’s expression and clinical traits of BLCA patients was assessed using independent sample *t*-test. The KEGG and GO enrichment analyses were performed with “clusterProfiler”. The WGCNA was carried out with “WGCNA”, the random forest was carried out with “randomforestSRC”, and the Univariate and multivariate Cox proportional hazards regression survival analyses were carried out with “survival”. The K-M survival curves were plotted with “survival”, the ROC curves were plotted with “survivalROC”, and the nomogram was plotted with “rms”. The *P*-value of less than 0.05 was considered as statistically significant.

## Results

### DEGs and enrichment analysis

**Fig. 1:**
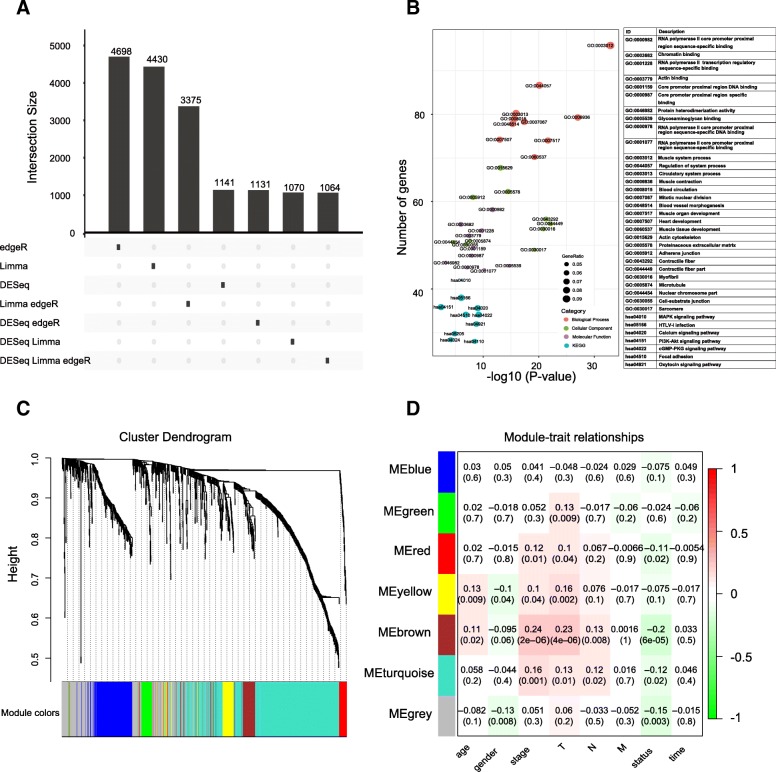
Enrichment analysis and weighted correlation network analysis for differentially expressed genes (DEGs). **a** An UpSet plot illustrating the overlaps among DEGs identified by edgeR, Limma, and DESeq. **b** Bubble plots for enriched GO and KEGG terms. The x-axis represents the -log10*P*-value) of each term and the y-axis represents the number of genes in each term. **c** Dendrogram generated using the WGCNA. **d** PCC matrix between MEs and clinical traits. The PCC values range from − 1 to 1 depending on the strength of the relationship. A positive value indicates that the genes within a particular co-expression module increase as the clinical trait increases, whereas the opposite is true if the PCC is negative. Each PCC value is accompanied by the corresponding *P*-value in brackets

The pre-processing step obtained 19,181 mRNAs. Furthermore, a total of 1064 overlapping DEGs were identified by “limma”, “edgeR”, and “DESeq”, with 242 up-regulated DEGs and 822 down-regulated DEGs. An UpSet plot indicating overlapping DEGs was presented in Fig. 1a. GO and KEGG functional enrichment analysis were used to detect the biological mechanism of DEGs in BLCA. According to the results in Fig. 1b and Additional file 4, the DEGs were most significantly enriched in transcription factor activity RNA polymerase II core promoter proximal region sequence-specific binding of GO Molecular Function, muscle system process of GO Biological Process, actin cytoskeleton of GO Cellular Component, and MAPK signaling pathway of KEGG.

### Identification of gene co-expression network and protein-protein interactions

**Fig. 2:**
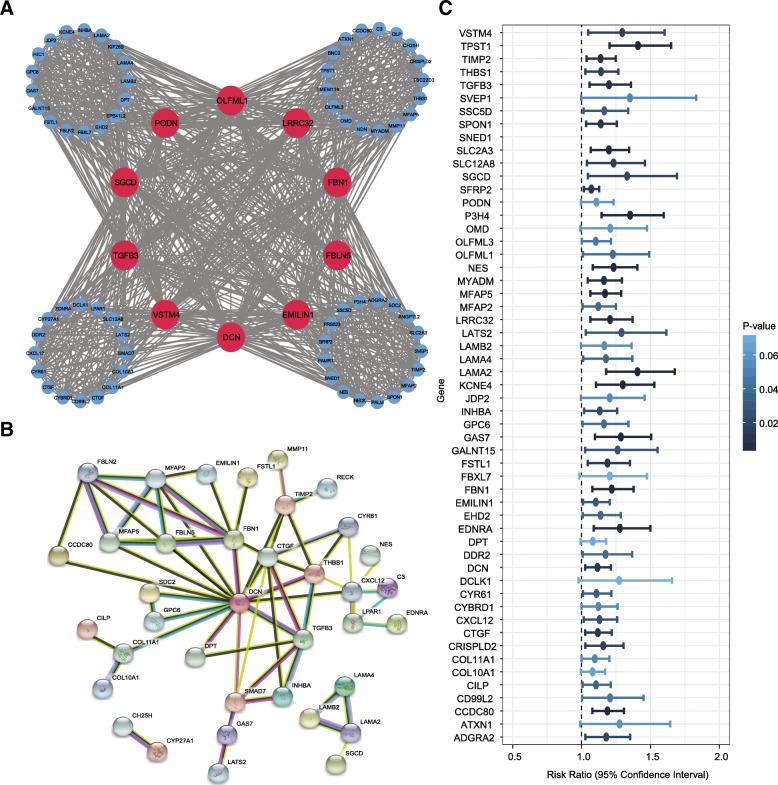
Co-expression network, protein-protein interactions (PPIs) and univariate survival analysis for 77 genes in the brown module. **a** The co-expression network for 77 genes in the brown module associated with the pathological stage. The red nodes represent hub genes obtained by WGCNA. **b** STRING database identified that 37 genes compactly interacted with each other. **c** A forest plot illustrating the independent prognostic value of 77 genes obtained from univariate Cox regression analysis. The points show the HRs and the corresponding lines show the 95% confidence intervals

According to scale independence and mean connectivity plot (Additional file 3: Figure S1A, B), we picked 5 as the proper soft-thresholding power which can raise co-expression similarity to achieve consistent scale-free topology. A total of seven modules that are highly co-expressed and ranged in size from 45 to 470 genes were identified (Fig. 1c). Each co-expression module was assigned by an arbitrary brilliant color for reference, the non-co-expression group was designated as a gray color. A topological overlap matrix (TOM) heatmap plot for co-expression modules was shown in Additional file 3: Figure S1C. The details of GO functional enrichment analysis for each module were provided in Additional file 5. The module eigengenes (MEs) based on the first principal component were calculated for each module to assess the association between modules and clinical information, and the corresponding module-clinical trait correlation was visualized as a heatmap plot (Fig. 1d). The results showed that the brown module possessed the highest correlation with pathological stage (*r* = 0.24, *P* < 0.01). A heatmap plot for Pearson’s correlation coefficient (PCC) of 77 genes in the brown module was provided in Additional file 3: Figure S1D. We further attempted to identify hub genes in the brown module. As a result, DCN, OLFML1, FBN1, SGCD, EMILIN1, PODN, LRRC32, TGFB3, VSTM4, and FBLN5 were identified as hub genes and the corresponding co-expression network was visualized with Cytoscape software (Fig. 2a).

**Fig. 3:**
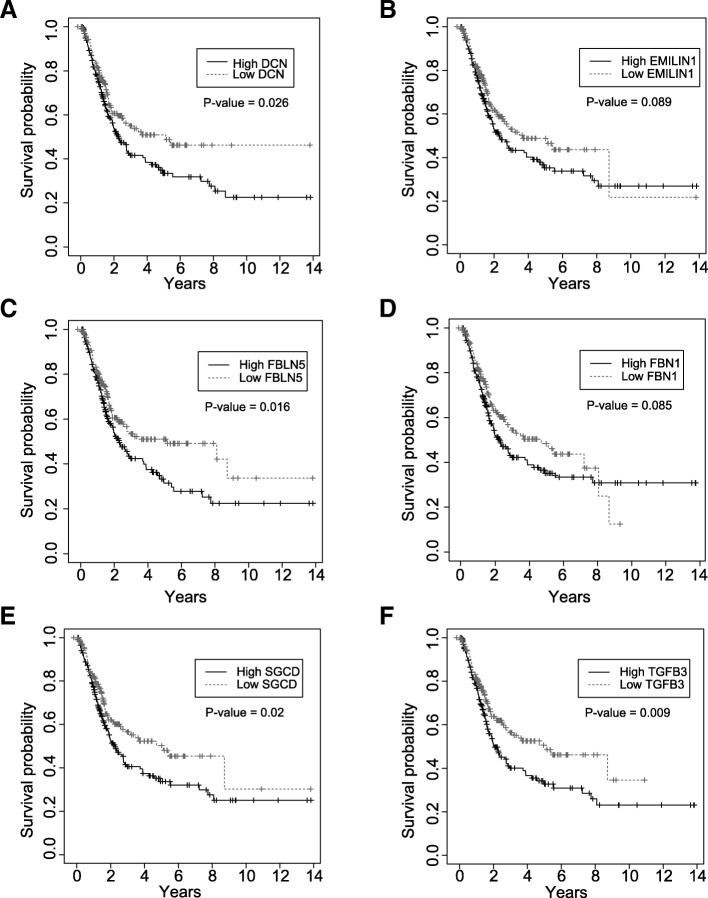
Kaplan-Meier survival plots for DCN, EMILIN1, FBLN5, FBN1, SGCD, and TGFB3. **a**-**f** The Kaplan-Meier survival curve revealed that ﻿high DCN, FBLN5, SGCD, and TGFB3 expression conferred the worse overall survival in patients with BLCA (*P* < 0.05)

In addition, the protein-protein interactions (PPIs) of 77 genes were examined using the STRING database. We found that 37 genes formed a complex functional network, indicating that each of them has at least one functionally similar or interacted gene as the neighbor (Fig. 2b). Remarkably, we found that six hub genes (DCN, EMILIN1, FBLN5, FBN1, SGCD, and TGFB3) obtained by the WGCNA tended to be in the central hub of the network generated using the STRING database, thereby demonstrating the importance of these genes and the accuracy of our method. We also evaluated the prognostic significance of six hub genes in patients with BLCA. The K-M survival analysis revealed that the higher expression level of DCN, FBLN5, SGCD, and TGFB3 was associated with the worse overall survival, suggesting that they may play an oncogenic role (Fig. 3a-f).

### Identification of prognostic biomarkers and construction of risk score model

**Fig. 4:**
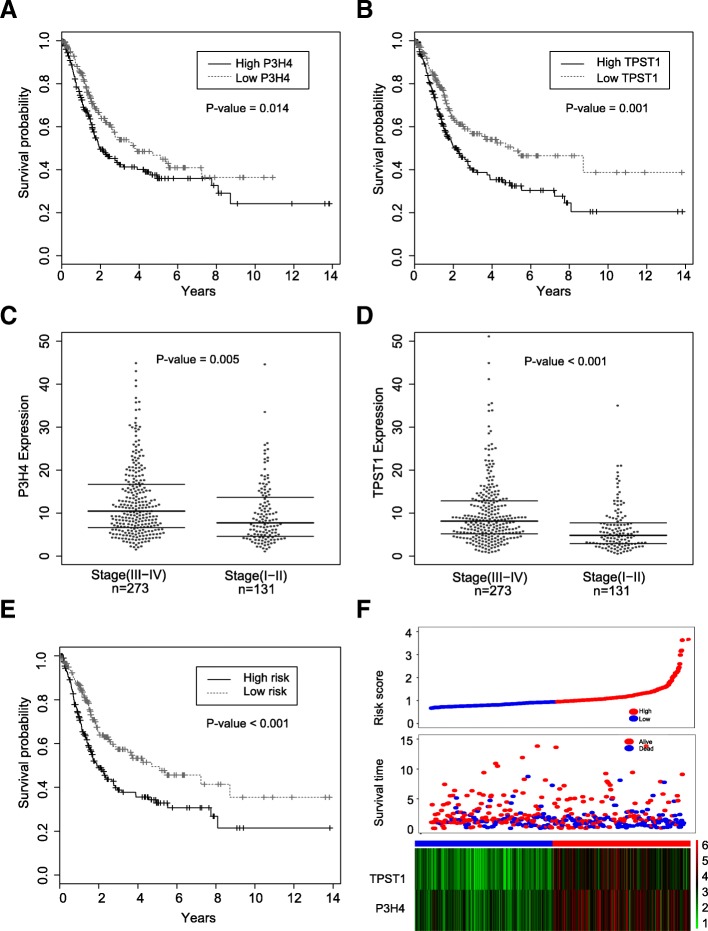
Construction of a risk score model based on the expression level of P3H4 and TPST1. **a**, **b** Kaplan-Meier survival plots for P3H4 and TPST1. High expression of P3H4 and TPST1 indicated a poorer prognosis. **c, d** The scattered plots for the expression level of P3H4 and TPST1 across different pathological stages in the TCGA dataset. Horizontal lines represent medians and dispersions. **e** Kaplan-Meier survival plots for high and low risk score groups in the TCGA dataset. **f** The detail information of the low and high score groups in the TCGA dataset (upper); the survival status and survival time of TCGA cohort (middle); heatmap for the TPST1 and P3H4 expression in the TCGA dataset, the color from green to red shows a trend from the low expression to the high expression (lower)

We carried out a univariate Cox regression analysis of 77 genes within the brown module to investigate the independent prognostic value. As a result, 43 genes were identified and the hazard ratios (HRs) and 95% confidence intervals of each gene were presented in Fig. 2c. TPST1, LAMA2, P3H4, CCDC80, and MFAP5 were the highest ranked risk markers as presented in Additional file 6. It is worth noting that the coefficients of 43 signatures were more than *1*, indicating 43 independent prognostic biomarkers are risk indicators. RF and multivariate Cox regression analysis were performed on 43 biomarkers to identify an optimal combination of biomarkers and to construct a risk score model. As a result, TPST1 and P3H4 were identified as an optimal combination of independent prognostic factors. The K-M survival curve revealed that ﻿high TPST1 or P3H4 expression conferred worse overall survival (Fig. 4a, b). The independent t-test analysis showed that the high pathological stage group was characterized by high expression of P3H4 and TPST1 (Fig. 4c, d). Given that their high association with pathological stage and survival, P3H4 and TPST1 were therefore chosen as the prognostic signatures for developing a risk score model. The risk score of each patient sample was calculated as the following: Risk Score = 0.029116 * TPST1 + 0.018074 * P3H4. As expected, the K-M survival curve revealed that patients with high risk score were correlated with worse overall survival (Fig. 4e). The detailed risk score, survival information and gene expression level of two biomarkers were shown in Fig. 4f**.**

**Fig. 5:**
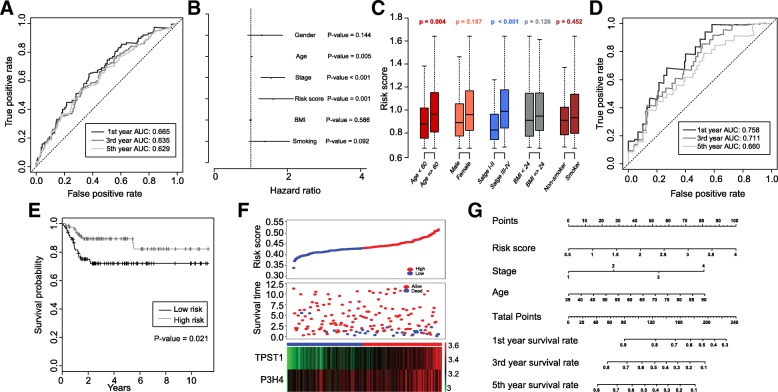
Performance of the risk score model in the training dataset and testing dataset. **a** ROC curve estimating the performance of the risk score model predicting first, third, and fifth-year survival in the TCGA dataset (training dataset). **b** Multivariate Cox regression analysis for the risk score model and other clinical traits. Hazard ratios, 95% confidence intervals, and *P*-value were displayed. **c** The correlation between the risk score and other clinical traits were calculated via independent t-test, corresponding *P*-values were shown at the top. **d** ROC curve estimating the performance of the risk score model predicting first, third, and fifth-year survival in the GSE13507 dataset (testing dataset). **e** Kaplan-Meier survival plots for high and low risk score groups in the GSE13507 dataset. **f** The detail information of the low and high score groups in the GSE13507 dataset (upper); the survival status and time of GSE13507 cohort (middle); heatmap for the TPST1 and P3H4 expression in the GSE13507 dataset, the color from green to red shows a trend from the low expression to the high expression (lower). **g** A prognostic nomogram for predicting first, third and fifth-year survival rate was delineated

The AUC revealed that the performances of the risk score model for prediction of first, third and fifth-year survival rate in the TCGA dataset reached 0.665, 0.635, and 0.629, respectively (Fig. 5a). The multivariate Cox proportional hazard model was employed for assessment of the impact of clinical indicators and risk score. As shown in Fig. 5b, the risk score model showed the highest hazard ratio (HR) of 1.59, with a 95% confidence interval ranged from 1.2128 to 2.165, suggesting the risk score model may have a higher BLCA prognostic effect than other indicators including the pathological stage. Remaining covariate clinical traits, like gender, BMI (Body Mass Index), and smoking history failed to attain statistical significance (*P* > 0.05). Besides, the correlation analyses between risk score and clinical indicators showed the risk score model was significantly associated with pathological stage and age (*t*-test, *P* < 0.05), while independent from BMI, gender and smoking history (Fig. 5c**).**

### Validation of risk score model and development of nomogram

We evaluated the performance of the risk score model on an independent microarray dataset GSE13507. The results showed that the AUC values of the risk score model for one-year survival rate, three-year survival rate, and five-year survival rate were 0.758, 0.711, and 0.66, respectively. (Fig. 5d). It was found that our proposed model significantly outperformed the previous models through comparative analyses (Table 1). K-M survival curves showed that the survival rate of the high risk group was significantly lower than that of the low risk score group (Fig. 5d) and patients with high risk score can be characterized as high expression of TPST1 and P3H4 (Fig. 5e), which together were in close agreement with the results observed in the TCGA dataset, suggesting again the importance of the risk score model in prognosis prediction of BLCA. At last, based on the risk score model, pathological stage, and age, we delineated a nomogram to predict the first, third and fifth-year survival rate. In accordance with the results of multivariate Cox proportional hazard model, the risk score model contributed the most risk points ranged 0 to 100, whereas the other clinical information contributed much less (*C*-index = 0.67; Fig. 5f).

**Table 1:** A comparison of our proposed model to other models

Author (Year)	Ref.	Predicted survival rate (Year)	AUC/samples (Training dataset)	AUC/samples (Testing dataset)	Number of genes	Genes
Our study	NA	3	0.635/*n* = 404	0.711/*n* = 165	2	P3H4; TPST1
H. Zhou et al. (2015)	[36]	5	0.74/84	NA/NA	8	miR-141-3p; miR-200c-3p; miR-21-5p; miR-145-5p; miR-125b-5p; miR-199a-5p; let-7c; miR-99a-5p
F. Peng et al. (2017)	[37]	5	0.664/*n* = 189	0.681/*n* = 188	3	hsa-mir-337; hsa-mir-3913-2; hsa-mir-497
Q. Liu et al. (2017)	[38]	3	0.647/NA	NA/NA	3	RCOR1; ST3GAL5; COL10A1
C. Liu et al. (2018)	[39]	5	0.83/*n* = 119;	0.68/*n* = 120	6	ACADS; C1QTNF9B; RP11-60 L3.1; CTD-3195I5.3; has-miR-3913-1; has-miR-891a
J. Chu et al. (2018)	[40]	3	0.615/ *n* = 407	NA/ NA	7	ZNF230; BCL2L14; AHNAK; TMEM109; APOL2; AGER; AOC2
Z. Xu et al. (2019)	[41]	NA	0.735/ *n* = 412	NA/ NA	11	MXRA7; EMP1; TNFA1P8L3; SERPINB12; SAPCD1; GABRG1; PLEKHG4B; ABCA4; PTPRR; XAGE2; PEX5L

Note: NA represents “not available”, Ref. represents References

## Discussion

The recent study investigated the gene co-expression network related to the pathological stage of BLCA and presented a risk score model based on the expression level of TPST1 and P3H4. The model performed well in predicting the one-year, three-year, and five-year survival rate of patients with BLCA.

We used the RNA-Seq data of BLCA downloaded from the TCGA to obtain 1064 differentially expressed genes, which were analyzed by the WGCNA to identify the brown co-expression module comprised of 77 pathological stage-specific genes. We observed that six genes (DCN, EMILIN1, FBLN5, FBN1, SGCD, and TGFB3) were hub genes in the brown co-expression module and were also located at the central hub of the PPI network generated using the STRING database, indicating that they may play an important role in the tumorigenesis and progression of BLCA. Lushun et al. have reported that FBN1, COL3A1, COL5A2, and POSTN were hub genes in both co-expression module and PPI network in bladder cancer [27]. Hu et al. found that the expression level of FBLN5 was downregulated in human bladder carcinoma samples, resulting in increased proliferation and invasiveness [28]. It is worth mentioning that, CXCL12, THBS1, MMP11, RECK, and TIMP-2 within the brown module also have an effect on the progression of BLCA and prognosis of patients with BLCA [29].

By employing Cox proportional hazard regression models integrated with random forest, we identified TPST1 and P3H4 as prognostic indicators. Further investigation found that the elevated TPST1 or P3H4 expression were significantly associated with poor survival and high tumor pathological stage. Moreover, we developed a TPST1 and P3H4-based risk score model, which outperforms the pathological stage and previously proposed models in the prediction of the prognosis of patients with BLCA. The group with high risk score was characterized by the high pathological stage and poor survival in the TCGA and GSE13507 datasets. It has been reported that TPST1 is overexpressed in bladder cancer, oral squamous cell cancer, breast cancer and barretina sarcoma [30–32] and involved in the invasion and metastasis of head and neck carcinoma and nasopharyngeal carcinoma [33, 34]. Although P3H4 has been reported as a tumor-associated auto-antigen in patients with prostate cancer, its biological functions in other cancers remain elusive [35].

The approach combining the WGCNA with the Cox Proportional-Hazards Model and random forest in this study has achieved reliable results regarding the survival-related co-expression network identification and risk score model construction. It is worth noting that we can enhance our study in the following aspects: (i) The performance of risk score model was estimated based on bioinformatics approaches, so the accuracy was affected by the data pre-processing, threshold criteria and statistical methods. Therefore, the application of the risk score model still needs to be verified in large cohort studies. (ii) Further studies are needed to elucidate the mechanism how hub genes in the brown module mutually interact and further influence BLCA progression. (iii) The functional analysis of P3H4 and TPST1 is of great value in understanding pathogenesis, guiding medications and treatment regimens, and analyzing drug resistance and prognosis, and it will be our next focus.

## Conclusion

In conclusion, we identified the gene co-expression module associated with pathological stage and investigated the underlying hub genes. Besides, we identified prognostic-related biomarkers and incorporated them into a risk score model for estimation of BLCA prognosis. Our findings will aid in a deeper understanding of the tumorigenesis and progression of BLCA. The risk score model we proposed may have important clinical value to the prognosis of patients with BLCA.

## Additional files

Additional file 1: **Table S1.** Baseline characteristics of the TCGA cohort. (DOCX 15 kb)

Additional file 2: **Table S2.** Baseline characteristics of the GSE13507 cohort. (DOCX 17 kb)

Additional file 3: **Figure S1.** Detailed information on WGCNA of DEGs. (**A, B**) Analysis of the scale-free fit index and mean connectivity for various soft-thresholding powers. (**C**) A topological overlap matrix (TOM) heatmap for seven co-expression modules. Hierarchical clustering was applied to the TOM-based dissimilarity matrix to identify modules. Light color represents low topological overlap and progressively darker red color represents higher overlap. Each module is assigned by a unique color; these are shown along the left side and the top. (**D**) A heatmap plot for PCC for 77 genes in the brown module. (PDF 6946 kb)

Additional file 4: KEGG and GO enrichment analyses for 1064 DEGs. (XLS 378 kb)

Additional file 5: The details of GO functional enrichment analysis for each module. (XLS 51 kb)

Additional file 6: Univariate survival analysis identified 43 genes as independent prognostic biomarkers. (XLS 20 kb)

## Data Availability

The authors declare that the data supporting the findings of this study are available within the articles.

## Abbreviations

AUC: Area under ROC curve
BLCA: Urothelial bladder cancer
BMI: Body Mass Index
DEGs: Differentially expressed genes
GEO: Gene Expression Omnibus
GO: Gene Ontology
HR: Hazard ratio
KEGG: Kyoto Encyclopedia of genes and Genomes
MEs: Module eigengenes
OS: Overall survival
PCC: Pearson’s correlation coefficient
RF: Random forest
TCGA: The Cancer Genome Atlas
TMM: Trimmed Mean of M values
TOM: topological overlap matrix
WGCNA: Weighted gene co-expression network analysis
