# Supplementary material for: A co-expression network for differentially expressed genes in bladder cancer and a risk score model for predicting survival
Source: Hereditas. 2019 Jul 9;156:24. doi: 10.1186/s41065-019-0100-1 (PMC6617625; doi:10.1186/s41065-019-0100-1)
Supplement: Supplementary file 1 — Table S1. Baseline characteristics of the TCGA cohort. (DOCX 15 kb) [file 41065_2019_100_MOESM1_ESM.docx]

**Table. S1. Baseline characteristics of the TCGA cohort.**

| **Characteristics** | **Group** | **Number of sample (%)** |
| --- | --- | --- |
| Recurrence | Recurred  Disease-free | 170 (42%)  234 (58%) |
| Gender | Male  Female | 297 (73%) 107 (27%) |
| Stage | Stage I  Stage II  Stage III  Stage IV | 2 (1%)  129 (32%)  138 (34%) 135 (33%) |
| Grade | High  Low | 384 (95%)  20 (5%) |
