# Supplementary material for: A co-expression network for differentially expressed genes in bladder cancer and a risk score model for predicting survival
Source: Hereditas. 2019 Jul 9;156:24. doi: 10.1186/s41065-019-0100-1 (PMC6617625; doi:10.1186/s41065-019-0100-1)
Supplement: Supplementary file 2 — Table S2. Baseline characteristics of the GSE13507 cohort. (DOCX 17 kb) [file 41065_2019_100_MOESM2_ESM.docx]

**Table. S2. Baseline characteristics of the GSE13507 cohort.**

| **Characteristics** | **Group** | **Number of sample (%)** |
| --- | --- | --- |
| Gender | Male  Female | 135 (82%) 30 (18%) |
| Invasiveness | Superficial  Invasive | 103 (63%)  62 (37%) |
| Chemotherapy | No  Yes | 138 (84%)  27 (16%) |
| Grade | Low  High | 105 (64%)  60 (36%) |
| Overall survival | Alive  Death | 96 (58%)  69 (42%) |
| T | T1  T2  T3  T4  Unknown | 80 (49%)  31 (20%) 19 (11%)  11 (6%)  24 (14%) |
| N | N0  N1  N2  N3  Unknown | 149 (90%)  8 (4%)  6 (2%)  1 (2%)  1 (2%) |
| M | M0  M1 | 158 (96%)  7 (4%) |
