# Supplementary figures and images for: A co-expression network for differentially expressed genes in bladder cancer and a risk score model for predicting survival
Source: Hereditas. 2019 Jul 9;156:24. doi: 10.1186/s41065-019-0100-1 (PMC6617625; doi:10.1186/s41065-019-0100-1)

**A**

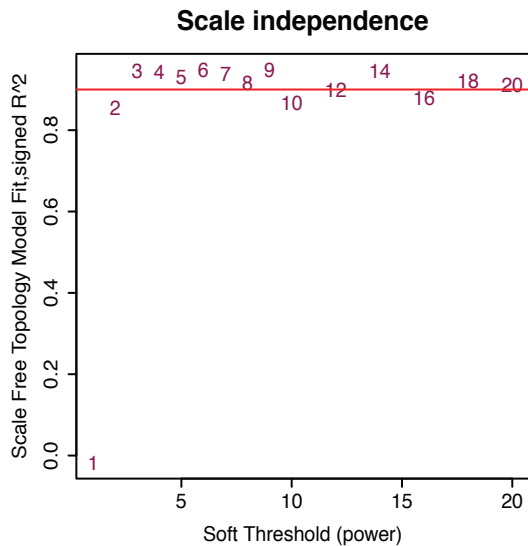

**C**

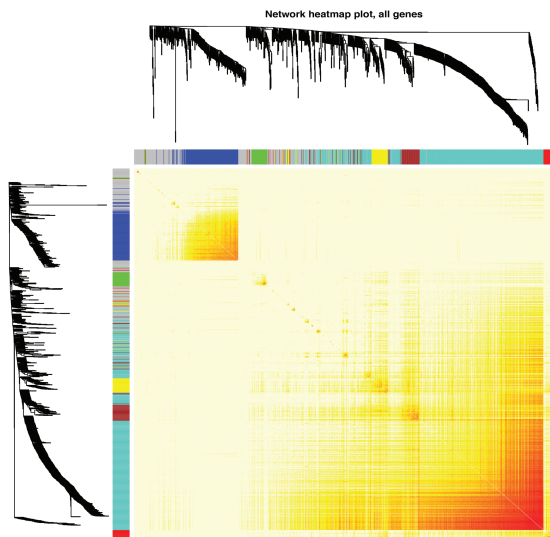

## B

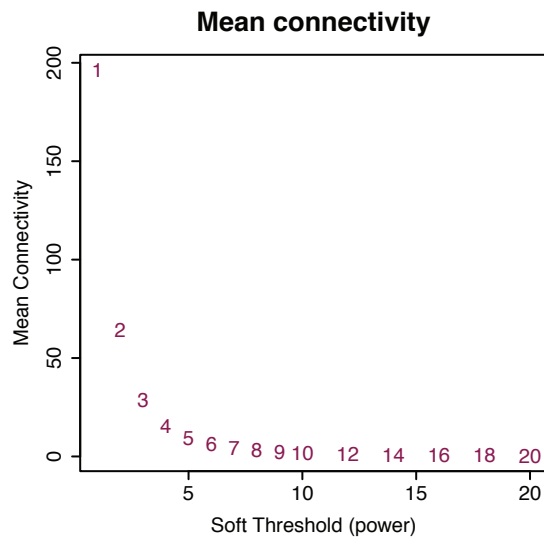

D

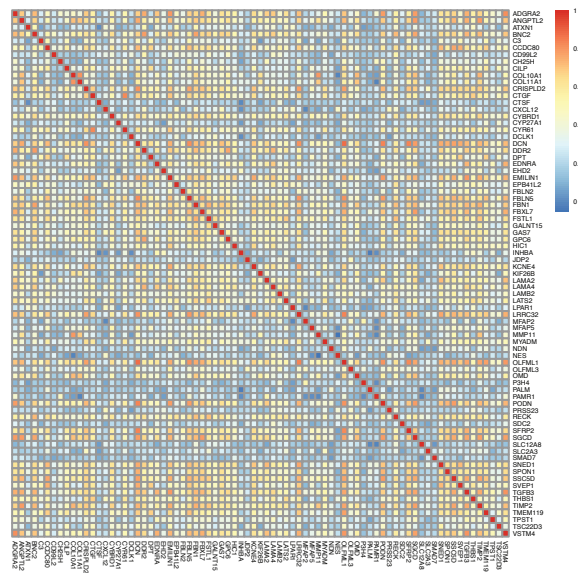

Supplement: Supplementary file 3 — Figure S1. Detailed information on WGCNA of DEGs. (A, B) Analysis of the scale-free fit index and mean connectivity for various soft-thresholding powers. (C) A topological overlap matrix (TOM) heatmap for seven co-expression modules. Hierarchical clustering was applied to the TOM-based dissimilarity matrix to identify modules. Light color represents low topological overlap and progressively darker red color represents higher overlap. Each module is assigned by a unique color; these are shown along the left side and the top. (D) A heatmap plot for PCC for 77 genes in the brown module. (PDF 6946 kb) [file 41065_2019_100_MOESM3_ESM.pdf]
